# Does Adding Electroanalgesic Modalities to a Multimodal Therapeutic Program Improve Clinical Outcomes in Individuals With Chronic Nonspecific Neck Pain? A Randomised Controlled Trial

**DOI:** 10.1002/ejp.70121

**Published:** 2025-09-05

**Authors:** Gabriela Nascimento de Santana, Aron Charles Barbosa da Silva, Patrícia Gabrielle dos Santos, Carlos Eduardo Girasol, Adriano Rodrigues de Oliveira, Almir Vieira Dibai‐Filho, Cid André Fidelis de Paula Gomes

**Affiliations:** ^1^ Postgraduate Program in Rehabilitation Sciences Nove de Julho University São Paulo Brazil; ^2^ Department of Physics, Faculty of Philosophy, Sciences and Letters University of São Paulo São Paulo Brazil; ^3^ Postgraduate Program in Rehabilitation and Functional Performance Federal University of Maranhão São Luís Brazil

## Abstract

**Background:**

Chronic nonspecific neck pain (CNSNP) is a prevalent and complex condition. Although many studies have evaluated the effectiveness of transcutaneous electrical nerve stimulation (TENS), interferential current (IFC), therapeutic exercise (TE), and manual therapy (MT) individually, this study aimed to determine whether adding IFC and/or TENS to a Multimodal Therapeutic Intervention Program (MTIP) would produce better outcomes than the MTIP alone concerning functional capacity, pain intensity, pain catastrophising, kinesiophobia and overall perceived effect in individuals with CNSNP.

**Methods:**

Seventy‐five individuals with CNSNP were randomly assigned to one of three groups: MTIP, MTIP + IFC, or MTIP + TENS. Interventions were conducted over 8 weeks. Outcomes were assessed at baseline, post‐intervention, and at a 1‐month follow‐up. The primary outcome was the Neck Disability Index (NDI). Secondary outcomes included the Numeric Pain Rating Scale (NPRS) at rest and during movement, the Pain‐Related Catastrophizing Thoughts Scale (PRCTS), the Tampa Scale for Kinesiophobia (TSK), the Copenhagen Neck Functional Disability Scale (CNFDS), the WHO Disability Assessment Schedule (WHODAS 2.0), and the Global Perceived Effect Scale (GPES).

**Results:**

No significant differences were observed between groups for the primary outcome. For secondary outcomes, the MTIP group showed improved results for NPRS‐m and CNFDS. Additionally, MTIP was superior to MTIP + IFC for NPRS‐r post‐intervention, while MTIP + TENS outperformed both groups at follow‐up. No significant differences were found for GPES, and none of the differences reached clinical significance.

**Conclusions:**

The addition of IFC and/or TENS to an MTIP did not enhance clinical outcomes in individuals with CNSNP.

**Significance:**

The results of this study assist clinicians in making informed decisions regarding the selection of therapeutic resources for managing chronic nonspecific neck pain. They also support researchers in refining and conducting new studies focused on improving the implementation of multimodal intervention protocols. Additionally, these findings help individuals with chronic nonspecific neck pain better understand which interventions may be most appropriate to include in their rehabilitation process.

**Trial Registration:**

NCT05400486

## 1 Introduction

Among the electroanalgesic methods for electrical stimulation (ES), transcutaneous electrical nerve stimulation (TENS) and interferential current (IFC) are frequently used to treat chronic nonspecific neck pain (CNSNP) and other musculoskeletal disorders (Malfliet et al. 2017; Rampazo et al. 2022).

Transcutaneous electrical nerve stimulation (TENS) is a therapeutic modality characterised by the application of low‐frequency currents (1–200 Hz) with a typical pulse duration of 100–200 microseconds (μs) (Johnson et al. 2022). In contrast, interferential current therapy (IFC) employs medium‐frequency alternating currents that intersect within the tissues, producing an amplitude‐modulated frequency typically ranging from 1 to 200 Hz (Almeida et al. 2018). Theoretically, IFC may achieve deeper tissue penetration and enhanced analgesic effects due to its modulated waveform and its ability to reduce skin impedance more effectively than low‐frequency currents (Johnson and Tabasam 2003). Nevertheless, these proposed benefits remain inadequately substantiated and lack consistent empirical support in the current literature (Almeida et al. 2018).

Theoretical support for ES modalities arises from their capacity to stimulate afferent sensory fibres, facilitating pain modulation through gate control and the endogenous opioid system (Hussein et al. 2022). TENS, in particular, has the potential to activate serotonin, cholinergic, and opioid receptors in the spinal cord, reducing primary hyperalgesia by stimulating GABA receptors (Beltran‐Alacreu et al. 2022).

Compared to the electroanalgesic modalities of ES, therapeutic exercise (TE) and manual therapy (MT) provide stronger evidence for their effectiveness in individuals with CNSNP (Calafiore et al. 2025). Based on high‐quality evidence and clinical practice guidelines, TE, particularly when incorporating resistance training, motor control, or mindfulness‐based approaches, is considered the first‐line intervention for reducing pain and improving function in CNSNP (Aguayo‐Alves et al. 2024; Mueller et al. 2023). Furthermore, combining TE and MT into a Multimodal Therapeutic Intervention Program (MTIP) has emerged as the most effective strategy for enhancing disability and reducing pain intensity. This approach has shown the highest probability (91%) of being the most effective first‐line treatment for individuals with CNSNP (Calafiore et al. 2025; Celenay et al. 2016).

Electroanalgesic modalities of ES, combined with TE and MT, are commonly used to manage CNSNP. Given their high prevalence and multifactorial nature, it is crucial to investigate the effectiveness of combining these approaches. TENS and IFC may serve as valuable adjuncts to TE and MT by enhancing functional outcomes and promoting adherence. However, current evidence is limited by heterogeneity and low methodological quality, highlighting the need for high‐quality randomised controlled trials (RCT) to evaluate their effects on outcomes beyond pain, such as disability and psychosocial factors.

Based on this rationale, the hypothesis of this study is that the combined use of TENS or IFC within a MTIP will lead to superior outcomes in functional capacity, pain intensity, pain catastrophising, kinesiophobia, and overall perceived effect in individuals with CNSNP. This study aimed to investigate whether the addition of IFC and/or TENS to an MTIP yields better outcomes compared to the MTIP alone, regarding functional capacity, pain intensity, pain catastrophising, kinesiophobia, and overall perceived effect in individuals with CNSNP.

## 2 Methods

### 2.1 Design

This is a single‐blind, superiority RCT with three parallel arms and blinded assessors, conducted from July 2022 to April 2025 at the Movement Laboratory of a private university in São Paulo, Brazil. The Human Research Ethics Committee of Nove de Julho University in São Paulo, Brazil (Protocol no. 55490521.3.0000.8069) approved the study, and the protocol was previously registered at clinicaltrials.gov (NCT05400486). All participants received both oral and written information before providing informed consent, which was obtained in both verbal and written forms. The study followed the Helsinki declaration and the guidelines established by the Consolidated Standards of Reporting Trials (Schulz et al. 2010).

### 2.2 Participants

Participants were recruited from the waiting list of a university rehabilitation clinic, two primary healthcare units, referrals from physiotherapists, and advertisements on flyers and posters displayed throughout the university campuses.

The sample included individuals of both sexes, aged 18 to 65 years, with CNSNP, who had experienced neck pain for over 90 days and had a Neck Disability Index (NDI) score of ≥ 5 and a Numerical Pain Rating Scale (NPRS) score of ≥ 3, either at rest or during active cervical movement (Pillastrini et al. 2016; Walker et al. 2008).

Exclusion criteria included individuals with neck pain caused by nerve root involvement, assessed through clinical examinations of dermatomes, myotomes, and reflexes. Those with a history of spinal surgery, physiotherapy for neck pain within the last 3 months, significant spinal disorders, spinal fractures, inflammatory or infectious diseases, or contraindications to electroanalgesic currents were also excluded. Furthermore, individuals with rheumatic, metabolic, neurological, or cardiopulmonary conditions, artificial cardiac pacemakers, sensory deficits, skin changes (especially at the application site), or a history of tumours or cancer within the past 5 years were excluded.

### 2.3 Randomization and Concealed Allocation

The clinical research coordinator, who was solely involved in this phase of the study, handled the randomisation and conducted weekly monitoring for potential adverse effects related to the interventions. Any adverse effects observed were documented and confirmed as related to the study procedures.

A random sequence was generated using Microsoft Excel (Microsoft Corporation, Washington) and divided into 25 blocks of 3 codes, ensuring a 1:1:1 allocation ratio among the MTIP, IFC + MTIP, and TENS + MTIP groups. The random codes were placed in opaque, sealed envelopes, sequentially numbered from 1 to 75, to maintain the confidentiality of participant assignments to the study groups.

Three investigators, each responsible for conducting procedures for one group and with over 15 years of experience in managing chronic musculoskeletal pain, were specially trained for this study and were solely responsible for assessing the participants. The training aimed to ensure the full implementation of all study protocols and was conducted in the same laboratory where the study took place, 2 months before participant recruitment began. Blinding both participants and physiotherapists was not feasible due to the nature of the interventions. During the initial session, participants were informed about the interventions they would be assigned to. Sessions occurred in a private room within the university laboratory, with 30‐min intervals in between to reduce contact among participants. Additionally, participants were advised not to discuss the interventions or procedures with anyone else.

## 3 Interventions

Participants were randomly assigned to one of three groups: MTIP, IFC + MTIP, or TENS + MTIP, with the interventions conducted over an 8‐week period. Sessions occurred twice a week on non‐consecutive days, each lasting approximately 90 min. All equipment used in the study was calibrated and complied with established technical standards.

In the first session, all groups participated in a 60‐min therapeutic intervention focused on pain education. The content was delivered through verbal and visual methods, utilising materials from Retrain Pain Foundations (Retrain Pain Foundation 2025). Topics included the pathophysiological mechanisms of chronic neck pain, coping strategies for pain management, reducing hypervigilance, and addressing beliefs and myths related to chronic neck pain, including misconceptions about imaging findings and treatment options.

### 3.1 MTIP

The MTIP protocol (Table S1) consisted of an 8‐week intervention divided into two 4‐week phases. It included manual therapy techniques such as mobilisation applications, combined with a structured therapeutic exercise programme aimed at stabilising the cervical spine and enhancing scapulothoracic stability (Barbosa da Silva et al. 2024). Therapeutic exercises with resistance were performed in three sets of 8 to 12 repetitions or held for 15 to 30 s each, with 120 s of rest between sets (Barbosa da Silva et al. 2024; Pillastrini et al. 2016). Elastic bands (Thera‐Band, The Hygenic Corporation, Akron, OH, USA) provided five colour‐coded resistance levels, where lighter colours indicated lower resistance and darker colours indicated higher resistance. For bodyweight exercises, a holding time of 30 s to 1 min was recommended, along with three repetitions. During manual therapy, the physical therapist performed three sets of 1‐min oscillatory mobilisations. Exercise progression was evaluated weekly and was based on the ability to complete 12 pain‐free repetitions of each exercise (Barbosa da Silva et al. 2024; Pillastrini et al. 2016).

### 3.2 IFC + MTIP

Immediately after the MTIP intervention, the IFC was applied using the Endophasys device (NMS‐0501, KLD Biossistemas Equipamentos Eletrônicos Ltda). Participants were seated with their necks exposed. Four self‐adhesive electrodes (5 × 5 cm) were arranged in a crossed pattern, with two positioned in the upper cervical area and two in the lower, forming a square around the centre of the neck. The following parameters were set: a carrier frequency of 4 kHz, a sweep mode of 1/1 s, an amplitude‐modulated frequency (AMF) of 60 Hz, a delta AMF of 30 Hz, and an automatic vector mode. The stimulation intensity was adjusted to the motor sensory threshold, and the total application time was 30 min (Albornoz‐Cabello et al. 2019, 2021).

### 3.3 TENS + MTIP

Additionally, after the MTIP intervention, TENS was applied using the Endophasys device (NMS‐0501, KLD Biossistemas Equipamentos Eletrônicos Ltda). Participants were seated with their cervical regions exposed. Four adhesive electrodes (5 × 5 cm) were placed around the neck area. The stimulation parameters were as follows: a pulse width of 100 μs, a frequency of 100 Hz, and an intensity set to the maximum tolerable motor threshold. The application lasted for 30 min (Martins‐de‐ Sousa et al. 2023).

## 4 Outcome Measures

All assessments were conducted in person at three different time points: baseline, post‐intervention (after 8 weeks), and one‐month follow‐up (1‐moFU). They were carried out by two researchers responsible for the assessments who had no role in any earlier phase of the study. Both researchers brought over 15 years of experience in managing chronic musculoskeletal pain and completed 2 months of training at the same laboratory where the research occurred.

Initially, all participants completed a researcher‐developed questionnaire to collect demographic and clinical data.

The primary outcome was disability and functionality, assessed using NDI. The NDI evaluates how neck pain impacts an individual's ability to perform daily activities. It consists of 10 items, each focusing on a specific aspect of function affected by neck pain. Each item is scored on a scale from 0 (indicating no disability) to 5 (indicating complete disability), with higher scores reflecting greater levels of disability (MacDermid et al. 2009). The minimum clinically important difference (MCID) for the NDI is considered to be 7 points (MacDermid et al. 2009).

Secondary outcomes included pain intensity, evaluated using the Numeric Pain Rating Scale (NPRS), which measures pain experienced over the past 7 days (NPRS‐r) and during movement (NPRS‐m). The NPRS is an 11‐point scale ranging from 0 (‘no pain’) to 10 (‘worst imaginable pain’), with higher scores indicating greater pain intensity (Pool et al. 2007). MCID for the NPRS is considered to be 2.5 points (Pool et al. 2007).

Catastrophic thinking was evaluated using the Pain‐Related Catastrophizing Thoughts Scale (PRCTS). The PRCTS comprises nine items aimed at assessing thoughts and attitudes linked to pain catastrophising in individuals suffering from chronic pain. Each item represents a cognitive or emotional response associated with the perception of pain. Higher scores signify increased levels of pain‐related catastrophising (Junior et al. 2008).

The fear of movement was assessed using the Tampa Scale for Kinesiophobia (TSK). The TSK is a self‐report questionnaire that consists of 17 items evaluating beliefs about fear of movement, re‐injury, and the perceived negative consequences of physical activity (Mendes et al. 2024). Each item is scored on a 4‐point Likert scale, where higher scores indicate greater levels of kinesiophobia. The MCID for the TSK is considered 4 points (Dupuis et al. 2023).

Functional disability associated with neck pain was evaluated using the Copenhagen Neck Functional Disability Scale (CNFDS). The CNFDS consists of 15 items that assess various aspects of physical and social functioning related to chronic neck pain. Each item measures the extent to which neck pain affects daily activities. Higher scores indicate greater levels of functional disability (Barreto et al. 2022). The MCID for the CNFDS is recognised as 7 points (Hessam et al. 2024).

Functioning and disability were assessed using the World Health Organization Disability Assessment Schedule (WHODAS 2.0). The WHODAS is based on the International Classification of Functioning, Disability, and Health (ICF) and consists of 12 items related to common daily activities and behaviours (Katajapuu et al. 2020). Higher scores indicate greater impairment in functioning and higher levels of disability. The MCID is 9 points (Katajapuu et al. 2020).

The overall effect of the treatment was evaluated using the Global Perceived Effect Scale (GPES) during the post‐intervention period (8 weeks) and at the 1‐month follow‐up. The GPES uses an 11‐point scale ranging from −5 to +5, where negative values indicate a perceived worsening compared to the start of the treatment, while positive values denote a perceived improvement (de Fátima Costa Oliveira et al. 2014).

It is important to note that including the NDI, CNFDS, and WHODAS 2.0 was intentional, as each measures different facets of disability: the NDI evaluates neck‐specific disability, the CNFDS assesses functional limitations in daily activities, and the WHODAS 2.0 offers a broader assessment aligned with the ICF framework. Together, they provide a more complete understanding of participants' functional status.

## 5 Statistic Analysis

Using Ene software version 3.0 from the Autonomous University of Barcelona, the sample size was determined based on the primary outcomes of disability and functionality as assessed by the NDI. The MCID of 7 points between groups (MacDermid et al. 2009) and a standard deviation (SD) of 7 points (Pillastrini et al. 2016) were considered. The calculation assumed a statistical power of 80% and a significance level (alpha) of 5%, leading to an estimated sample size of 20 individuals per group. To account for a potential 5% dropout rate, a minimum of 21 participants was recruited per group.

An intention‐to‐treat analysis was conducted, establishing a significance level of 5% for all comparisons. The normality of the data was evaluated using histograms, which indicated that all variables exhibited a normal distribution. Descriptive statistics are reported as means and standard deviations (SD). Adjusted mean differences (MD) and 95% confidence intervals (CI) were calculated using linear mixed models that included fixed effects for group, time, and group‐by‐time interaction. Statistical analyses were performed using SPSS software, version 23.0 (IBM Corp., Armonk, NY, USA).

## 6 Results

**FIGURE 1:**
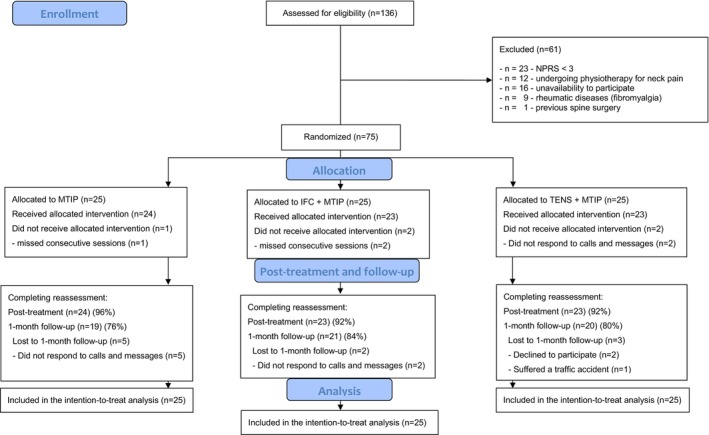
Flowchart of participants through the trial. NPRS: Numeric Pain Rating Scale; MTIP: Multimodal Therapeutic Intervention Program; MTIP + IFC: Multimodal Therapeutic Intervention Program + Interferential Current; MTIP + TENS: Multimodal Therapeutic Intervention Program + Transcutaneous Electrical Nerve Stimulation.

A total of 136 individuals were screened for eligibility, of which 75 met the inclusion criteria, consented to participate, and signed the informed consent form. The reasons for exclusion are detailed in Figure 1. Subsequently, the 75 participants were randomly allocated into three groups: MTIP (*n* = 25), MTIP + IFC (*n* = 25), and MTIP + TENS (*n* = 25). Specifically, 24, 23, and 23 participants completed the 8‐week follow‐up. At the 1‐month follow‐up, 19 participants in the MTIP group, 21 in the IFC + MTIP group, and 20 in the TENS + MTIP group completed the assessments (Figure 1).

At baseline, the groups displayed similar clinical and sociodemographic characteristics (Table 1). The study participants experienced moderate cervical functional disability according to the NDI and CNFDS, as well as mild to moderate functional impact based on WHODAS 2.0. Pain intensity was mild to moderate at rest and moderate to severe during movement. Pain catastrophising levels were low, while kinesiophobia ranged from low to moderate (Table 1). These findings suggest a sample with clinically significant cervical disability and pain, particularly during movement; however, pain catastrophising and kinesiophobia levels remain low.

**TABLE 1:** Baseline characteristics of the participants.

Variable	MTIP (*n* = 25)	IFC + MTIP (*n* = 25)	TENS + MTIP (*n* = 25)
Age (y), mean (SD)	37.40 (9.45)	37.20 (7.10)	38.88 (6.86)
Woman, *n* (%)	21 (84)	21 (84)	23 (92)
Weight (kg), mean (SD)	61.10 (11.19)	63.93 (12.56)	71.15 (5.46)
Height (cm), mean (SD)	1.57 (0.07)	1.60 (0.09)	1.64 (0.06)
BMI (kg/cm^2^), mean (SD)	24.82 (4.20)	24.99 (4.59)	26.21 (2.48)
Academic level, *n* (%)			
Less than 5 years of study	5 (20)	5 (20)	0 (0)
Completed 10 years of study	12 (48)	13 (52)	8 (32)
Completed 15 years of study	8 (32)	7 (28)	10 (40)
More than 15 years of study	0 (0)	0 (0)	7 (28)
Self‐reported physical activity practice, *n* (%)			
Yes	7 (28)	9 (36)	6 (24)
No	18 (72)	16 (64)	19 (76)
Diagnosis time (y), mean (SD)	4.72 (1.72)	5.04 (1.09)	4.52 (2.04)
NDI (0–50)	15.92 (2.17)	16.12 (3.23)	15.04 (2.31)
NPRS rest (0–10)	3.96 (0.97)	4.04 (0.73)	4.16 (0.94)
NPRS movement (0–10)	6.08 (0.99)	6.12 (1.01)	6.20 (0.95)
PRCTS (0–45)	0.75 (0.18)	0.69 (0.24)	0.76 (0.21)
TSK (1–68)	23.96 (3.58)	24.80 (3.50)	23.72 (2.83)
CNFDS (0–30)	16.80 (3.40)	16.92 (3.65)	17.04 (3.39)
WHODAS (1–60)	15.56 (2.04)	16.16 (2.05)	16.80 (2.39)

*Note:* Self‐reported physical activity practice (based on the question: Do you engage in physical activity?).

Abbreviations: CNFDS, the Copenhagen Neck Functional Disability Scale (high score = higher level of functional disability); IFC + MTIP, Multimodal Therapeutic Intervention Program + interferential current; MTIP, Multimodal Therapeutic Intervention Program; NDI, Neck Disability Index (high score = higher level of functional disability); NPRS, Numeric Pain Rating Scale (high score = higher level of pain intensity); PRCTS, Pain‐Related Catastrophizing Thoughts Scale (high score = higher level of catastrophizing); SD, standard deviation; TENS + MTIP, Multimodal Therapeutic Intervention Program + Transcutaneous Electrical Nerve Stimulation; TSK, Tampa Scale for Kinesiophobia (high score = higher level of Kinesiophobia); WHODAS, World Health Organization Disability Assessment Schedule (high score = higher level of disability and functioning); y, years.

Table 2 presents the means and standard deviations of outcome measures for participants in each group. Table 3 illustrates the within‐group comparisons from the post‐intervention to one‐month follow‐up periods.

**TABLE 2:** Mean (SD) of groups of study.

Outcomes	Groups								
	Baseline			Post			1‐moFU		
	MTIP (*n* = 25)	IFC + MTIP (*n* = 25)	TENS + MTIP (*n* = 25)	MTIP (*n* = 24)	IFC + MTIP (*n* = 23)	TENS + MTIP (*n* = 23)	MTIP (*n* = 19)	IFC + MTIP (*n* = 21)	TENS + MTIP (*n* = 20)
NDI (0–50)	15.92 (2.17)	16.12 (3.23)	15.04 (2.31)	13.62 (2.91)	14.04 (2.67)	13.42 (0.67)	13.37 (2.47)	13.29 (2.91)	11.95 (2.83)
NPRS‐r (0–10)	3.96 (0.97)	4.04 (0.73)	4.16 (0.94)	2.79 (0.97)	3.39 (0.94)	2.83 (1.02)	2.68 (0.88)	3.24 (0.88)	2.20 (0.89)
NPRS‐m (0–10)	6.08 (0.99)	6.16 (1.01)	6.20 (0.95)	3.96 (1.12)	5.04 (1.02)	4.91 (0.90)	3.53 (0.84)	4.81 (1.03)	4.59 (1.05)
PRCTS (0–45)	0.75 (0.18)	0.69 (0.24)	0.76 (0.21)	0.70 (0.18)	0.59 (0.22)	0.66 (0.20)	0.64 (0.13)	0.56 (0.18)	0.62 (0.23)
TSK (1–68)	23.96 (3.58)	24.80 (3.50)	23.72 (2.83)	23.33 (2.86)	23.91 (3.63)	22.70 (2.85)	23.05 (3.02)	23.43 (3.62)	23.10 (2.97)
CNFDS (0–30)	16.80 (3.40)	16.92 (3.65)	17.04 (3.39)	14.71 (3.11)	16.26 (4.33)	15.87 (3.27)	15.16 (3.50)	15.33 (4.29)	15.00 (3.01)
WHODAS (1–60)	15.56 (2.04)	16.16 (2.05)	16.80 (2.39)	14.79 (1.74)	15.13 (1.66)	15.70 (1.86)	14.05 (1.74)	14.52 (1.83)	15.26 (1.69)
GPES (−5 + 5)	—	—	—	2.33 (0.56)	2.48 (0.59)	2.48 (0.59)	2.32 (0.58)	2.33 (0.57)	2.37 (0.49)

Abbreviations: CNFDS, the Copenhagen Neck Functional Disability Scale (high score = higher level of functional disability); GPES, Global Perceived Effect Scale (− values indicate perceived worsening compared to the start of treatment, and + values indicate perceived improvement); IFC + MTIP, Multimodal Therapeutic Intervention Program + interferential current; MTIP, Multimodal Therapeutic Intervention Program; NDI, Neck Disability Index (high score = higher level of functional disability); NPRS‐m, Numeric Pain Rating Scale movement (high score = higher level of pain intensity); NPRS‐r, Numeric Pain Rating Scale rest (high score = higher level of pain intensity); PRCTS, Pain‐Related Catastrophizing Thoughts Scale (high score = higher level of catastrophizing); TENS + MTIP, Multimodal Therapeutic Intervention Program + Transcutaneous Electrical Nerve Stimulation; TSK, Tampa Scale for Kinesiophobia (high score = higher level of Kinesiophobia); WHODAS, World Health Organization Disability Assessment Schedule (high score = higher level of disability and functioning).

**TABLE 3:** Within‐group differences and mean (95% CI).

Outcomes	Within‐group differences					
	Post minus baseline			1‐moFU minus baseline		
	MTIP	IFC + MTIP	TENS + MTIP	MTIP	IFC + MTIP	TENS + MTIP
NDI (0–50)	−2.21* (−3.48 to −0.94)	−1.90* (−2.72 to −1.08)	−1.40* (−2.09 to −0.70)	−2.68* (−3.99 to 1.37)	−2.61* (−3.72 to −1.51)	−2.90* (−3.79 to −2.00)
NPRS‐r (0–10)	−1.21* (−1.89 to −0.52)	−0.66* (−1.15 to −0.17)	1.30*(−1.73 to −0.87)	−1.26* (−1.92 to −0.59)	−0.76* (−1.26 to −0.25)	−1.90* (−2.46 to −1.33)
NPRS‐m (0–10)	−2.15* (−2.70 to −1.61)	−1.09* (−1.60 to −0.58)	−1.27* (−1.79 to −0.75)	−2.63* (−3.21 to −2.05)	−1.38* (−1.93 to −0.082)	−1.59* (−2.09 to −1.08)
PRCTS (0–45)	−0.05 (−0.11 to 0.01)	−0.11* (−0.17 to −0.04)	−0.09* (−0.14 to −0.04)	−0.11* (−0.16 to −0.06)	−0.14* (−0.22 to −0.06)	−0.12* (−0.18 to −0.07)
TSK (1–68)	−0.57 (−1.28 to 0.12)	−0.95 (−1.99 to 0.09)	−0.65* (−1.23 to −0.07)	−0.47 (−1.26 to 0.31)	1.38* (−2.62 to −0.13)	−0.65* (−1.16 to −0.13)
CNFDS (0–30)	−2.05* (−3.27 to −0.83)	−0.42 (−1.64 to 0.78)	−1.15* (−1.77 to −0.54)	−1.73* (−3.11 to −0.35)	−1.61* (−2.54 to −0.69)	−1.73* (−2.61 to −0.86)
WHODAS (1–60)	−0.73* (−1.46 to −0.01)	−1.04* (−1.84 to −0.25)	−0.89* (−1.64 to −0.14)	−1.31* (−2.19 to −0.43)	−1.66* (−2.78 to −0.55)	−1.26* (−2.13 to −0.38)

Abbreviations: CNFDS, the Copenhagen Neck Functional Disability Scale (high score = higher level of functional disability); GPES, Global Perceived Effect Scale (− values indicate perceived worsening compared to the start of treatment, and + values indicate perceived improvement); MTIP, Multimodal Therapeutic Intervention Program; MTIP + IFC, Multimodal Therapeutic Intervention Program + interferential current; MTIP + TENS, Multimodal Therapeutic Intervention Program + Transcutaneous Electrical Nerve Stimulation; NDI, Neck Disability Index (high score = higher level of functional disability); NPRS‐m, Numeric Pain Rating Scale movement (high score = higher level of pain intensity); NPRS‐r, Numeric Pain Rating Scale rest (high score = higher level of pain intensity); PRCTS, Pain‐Related Catastrophizing Thoughts Scale (high score = higher level of catastrophizing); TSK, Tampa Scale for Kinesiophobia (high score = higher level of Kinesiophobia); WHODAS, World Health Organization Disability Assessment Schedule (high score = higher level of disability and functioning).

* *p* < 0.05.

No statistically significant differences were observed between groups in terms of disability and functionality as measured by the NDI, both at post‐intervention and at the one‐month follow‐up (Table 4).

**TABLE 4:** Between‐group differences and mean (95% CI).

Outcomes	Between‐group differences					
	Post minus baseline			1‐moFU minus baseline		
	MTIP vs. MTIP + IFC	MTIP vs. MTIP + TENS	MTIP + IFC vs. MTIP + TENS	MTIP vs. MTIP + IFC	MTIP vs. MTIP + TENS	MTIP + IFC vs. MTIP + TENS
NDI (0–50)	0.06 (−0.88 to 1.02)	0.78 (−0.16 to 1.74)	0.72 (−0.24 to 1.68)	−0.07 (−1.09 to 0.93)	−0.25 (−1.28 to 0.76)	−0.18 (−1.18 to 0.82)
NPRS‐r (0–10)	0.49* (0.01 to 0.97)	−0.17 (−0.65 to 0.30)	−0.66* (−1.15 to −0.18)	0.47 (−0.03 to 0.98)	−0.68* (−1.19 to −0.16)	−1.15* (−1.66 to −0.64)
NPRS‐m (0–10)	1.03* (0.57 to 1.50)	0.88* (0.41 to 1.34)	−0.15 (−0.62 to 0.31)	1.23* (0.74 to 1.73)	1.02* (0.53 to 1.51)	−0.21 (−0.70 to 0.26)
PRCTS (0–45)	−0.05 (−0.11 to 0.01)	−0.04 (−0.10 to 0.01)	0.01 (−0.05 to 0.06)	−0.02 (−0.08 to 0.03)	−0.01 (−0.07 to 0.05)	0.01 (−0.05 to 0.07)
TSK (1–68)	−0.17 (−0.92 to 0.58)	−0.10 (−0.85 to 0.64)	0.06 (−0.69 to 0.82)	−0.79 (−1.59 to 0.01)	−0.17 (−0.97 to 0.63)	0.61 (−0.17 to 1.41)
CNFDS (0–30)	1.35* (0.33 to 2.37)	0.87 (−0.14 to 1.89)	−0.48 (−1.51 to 0.54)	−0.01 (−1.09 to 1.07)	−0.03 (−1.14 to 1.06)	−0.02 (−1.11 to 1.06)
WHODAS (1–60)	−0.15 (−0.88 to 0.57)	−0.13 (−0.86 to 0.59)	0.01 (−0.72 to 0.75)	−0.25 (−1.03 to 0.52)	0.05 (−0.74 to 0.84)	0.30 (−0.47 to 1.08)

Abbreviations: CNFDS, the Copenhagen Neck Functional Disability Scale (high score = higher level of functional disability); GPES, Global perceived effect Scale (− values indicate perceived worsening compared to the start of treatment, and + values indicate perceived improvement); MTIP, Multimodal Therapeutic Intervention Program; MTIP + IFC, Multimodal Therapeutic Intervention Program + interferential current; MTIP + TENS, Multimodal Therapeutic Intervention Program + Transcutaneous Electrical Nerve Stimulation; NDI, Neck Disability Index (high score = higher level of functional disability); NPRS‐m, Numeric Pain Rating Scale movement (high score = higher level of pain intensity); NPRS‐r, Numeric Pain Rating Scale rest (high score = higher level of pain intensity); PRCTS, Pain‐Related Catastrophizing Thoughts Scale (high score = higher level of catastrophizing); TSK, Tampa Scale for Kinesiophobia (high score = higher level of Kinesiophobia); WHODAS, World Health Organization Disability Assessment Schedule (high score = higher level of disability and functioning).

* *p* < 0.05.

Regarding secondary outcomes, statistically significant differences were found for resting pain intensity (NPRS‐r), movement‐related pain intensity (NPRS‐m), and functional disability assessed by the CNFDS (Table 4). For NPRS‐r, significant differences were observed post‐intervention between MTIP and IFC + MTIP (in favour of MTIP), as well as between IFC + MTIP and TENS + MTIP (in favour of TENS + MTIP). At the 1‐month follow‐up, differences were noted between MTIP and TENS + MTIP, and between MTIP + IFC and TENS + MTIP (both in favour of TENS + MTIP). For NPRS‐m, significant differences were found at both post‐intervention and 1 month follow‐up between MTIP and IFC + MTIP, and between MTIP and TENS + MTIP, both in favour of the MTIP group. For CNFDS, a significant difference was observed post‐intervention between MTIP and IFC + MTIP, in favour of the MTIP group (Table 4). However, none of the statistically significant differences presented clinically meaningful results.

No statistically significant differences were found among the MTIP, MTIP + IFC, and MTIP + TENS groups in terms of the Global Perceived Effect (GPE). The adjusted mean difference between MTIP and IFC + MTIP was −0.10 points (95% CI: −0.42 to 0.21), between MTIP and TENS + MTIP it was −0.13 points (95% CI: −0.45 to 0.19), and between IFC + MTIP and TENS + MTIP, it was −0.02 points (95% CI: −0.34 to 0.29).

No adverse effects were reported by any of the participants throughout the study.

## 7 Discussion

This study is the first superiority RCT to investigate the additional effects of TENS and IFC when combined with an MTIP that includes ET and MT for individuals with CNSNP. The results indicate that neither TENS nor IFC offers extra benefits over MTIP alone in reducing disability for this group. Regarding secondary outcomes, the MTIP group showed better results for pain intensity during movement (NPRS‐m) and functional disability (CNFDS), while TENS only provided additional benefits for resting pain intensity (NPRS‐r). However, none of these differences were clinically meaningful.

Among the strengths of this RCT are its 8‐week intervention period and the inclusion of a 1‐month follow‐up, with dropout rates staying within acceptable limits for randomised trials (Cramer et al. 2016). The sample size was determined through a priori power analysis to detect clinically meaningful differences between groups. The study also maintained methodological rigour through true randomisation, allocation concealment, and intention‐to‐treat analysis. Importantly, all interventions were based on standard clinical practice guidelines (Blanpied et al. 2017) for managing individuals with CNSNP.

Although manual therapy was not included in one study's intervention protocol (Yesil et al. 2018), and neither therapeutic exercise nor manual therapy was applied in another (Acedo et al. 2015), the results from both trials can still be considered relevant comparisons to the present study.

Yesil et al. (2018) examined the effects of incorporating TENS and IFC into a therapeutic programme for individuals with chronic neck pain. Despite a brief intervention period of 3 weeks, the study found that including home‐based exercises, a lower stimulation frequency of 80 Hz for TENS, and a shorter exposure duration to electroanalgesic modalities produced results similar to those observed in this study. Specifically, adding TENS and/or IFC to a therapeutic exercise protocol did not lead to better outcomes than exercise alone.

Implementing a therapeutic protocol centered on therapeutic exercise is crucial for accurately evaluating the added value of TENS or IFC. Additionally, conducting follow‐up assessments beyond short‐term periods (e.g., 4 weeks) becomes important, especially in light of previous findings on the effects of electroanalgesic stimulation (Acedo et al. 2015). When these modalities are used as standalone interventions with brief follow‐up periods, both show statistically significant pain reductions, with IFC also showing clinically significant improvements (Acedo et al. 2015). However, as seen in the current study, over a longer period (8 weeks) and when incorporated into routine clinical practice as part of an MTIP, their isolated effects tend to decrease, providing no extra benefit compared to MTIP alone.

Indeed, some previous RCTs (Albornoz‐Cabello et al. 2019, 2021) suggest that including IFC in intervention protocols based on therapeutic exercise may lead to better outcomes for individuals with CNSNP. The addition of IFC is linked to improvements in pain intensity and disability (Albornoz‐Cabello et al. 2019, 2021). However, despite these positive results, the intervention periods in those studies lasted only 2 weeks, which is much shorter than the duration of the current trial. This raises concerns that the results may have been affected by timing bias, where short‐term assessments detect temporary effects that might not be sustainable over the long term.

A systematic review found evidence supporting the effectiveness of IFC and TENS for CNSNP (Rampazo et al. 2022), suggesting that both modalities may be beneficial as adjuncts to reduce pain and improve function in the immediate post‐treatment and short‐term periods. However, regarding disability (measured by the NDI), the primary outcome of the present study, as well as psychosocial factors, the quality of evidence was rated as low and very low, respectively. In light of these findings and those from the current trial, it appears that when longer follow‐up periods are used and methodological rigour is prioritised, the effects of these modalities may decrease in strength compared to immediate or short‐term outcomes. This is especially evident for functional disability, a domain already marked by significant uncertainty in previous evidence.

It is important to emphasise that, when discussing electroanalgesia, the stimulation parameters for both TENS and IFC must be carefully considered. In this study, the common parameters aligned with those reported in earlier clinical practice and research (de Espíndula Brehm et al. 2024). However, a high‐frequency protocol was chosen based on the frequency used. This highlights the need for future research to investigate low‐frequency stimulation, as high and low frequencies are associated with different physiological mechanisms. High‐frequency stimulation, as used here, is known to activate δ‐opioid receptors, enhance endogenous inhibitory pathways in the central nervous system involving GABA, muscarinic, and opioid receptors, increase β‐endorphin levels in blood and cerebrospinal fluid, reduce central neuronal sensitisation, and affect the release of excitatory neurotransmitters like glutamate and substance P in the dorsal horn of the spinal cord (de Espíndula Brehm et al. 2024).

The findings of this current RCT align with those reported in two recent systematic reviews with meta‐analyses (Cho et al. 2023; Mueller et al. 2023), confirming the effectiveness of therapeutic exercise, especially for individuals with CNSNP. The benefits are particularly evident when interventions are delivered more frequently and with longer session durations (Mueller et al. 2023). Clinicians and researchers should focus on developing multimodal intervention protocols that combine manual therapy and therapeutic exercise, as this approach has been linked to better clinical outcomes. These effects may be partly explained by evidence indicating that such combinations cause significant structural and functional changes in brain regions involved in pain perception and modulation, such as the anterior cingulate cortex and primary motor cortex (M1) (Chaikla et al. 2025).

This study may offer valuable insights into managing the application of therapeutic resources, such as multimodal therapy. Although the intervention dose and exposure time were selected to ensure effective treatment and maximise therapeutic benefits, delivering 90‐min sessions over 8 weeks could pose a challenge in routine clinical practice. Future research should explore the feasibility of shorter or more practical intervention schedules that better align with real‐world conditions, thereby enhancing external validity.

From a clinical perspective, this study indicates that routinely including TENS or IFC may needlessly extend treatment time, raise costs, and consume more resources without providing additional benefits in reducing disability. Therefore, the supplementary use of TENS and IFC should be carefully reconsidered, except in cases with specific indications for other outcomes or when first‐line treatments are not feasible. Our findings also support previous evidence showing that therapeutic exercise and manual therapy remain essential interventions for CNSNP (Cho et al. 2023; Mueller et al. 2023; Chaikla et al. 2025) and should be prioritised to optimise rehabilitation protocols and enhance outcomes for individuals with chronic nonspecific neck pain.

## 8 Limitation

The present study has several limitations. To reduce bias, outcome assessors were blinded, standardised protocols were followed, and validated self‐report measures were used. However, the interventions were delivered by three physical therapists who, due to the nature of the treatments, could not be blinded to group assignment. While the study aimed to mirror real‐world clinical conditions, where sham protocols are rarely used, it did not include a true control or sham intervention. Adding such a control could have improved internal validity and better isolated the specific effects of TENS/IFC. Additionally, participants were recruited only from public health facilities in one region of São Paulo, which may limit the applicability of the results to other populations and healthcare settings.

Despite these limitations, this study offers valuable insights that could inform future research. More trials with stricter designs and larger sample sizes are necessary to assess the long‐term effects of combining electroanalgesic methods with multimodal treatments in CNSNP. Future studies should also explore individual predictors of response to TENS and/or IFC and examine brain changes through neuroimaging.

## 9 Conclusion

Adding IFC and/or TENS into a multimodal treatment program that includes manual therapy and therapeutic exercises did not enhance outcomes related to functional capacity, pain catastrophising, kinesiophobia or overall perceived effect in individuals with CNSNP. Although TENS provided a slight additional benefit for resting pain, no results were clinically significant. Therefore, despite the study's limitations, it can be concluded that adding IFC and/or TENS appears unnecessary when using a multimodal protocol that combines manual therapy and therapeutic exercise over an 8‐week period.

## Author Contributions

This study was designed by G.N.d.S., A.V.D.‐F., and C.A.F.d.P.G. The experiments were performed by A.C.B.d.S., G.N.d.S., A.R.d.O., C.E.G., and P.G.d.S. Data validation and resource management were carried out by G.N.d.S., C.E.G., and P.G.d.S. Data were analysed by G.N.d.S., A.V.D.‐F., and C.A.F.d.P.G., and the results were critically examined by all authors. The original draft was primarily written by G.N.d.S., A.V.D.‐F., and C.A.F.d.P.G., and the manuscript was reviewed and edited by G.N.d.S., A.V.D.‐F., C.E.G., and C.A.F.d.P.G. was responsible for project supervision. All authors approved the final version of the manuscript and agree to be accountable for all aspects of the work.

## Supporting information


**Data S1:** ejp70121‐sup‐0001‐supinfo.docx.
