# Supplementary material for: Does Adding Electroanalgesic Modalities to a Multimodal Therapeutic Program Improve Clinical Outcomes in Individuals With Chronic Nonspecific Neck Pain? A Randomised Controlled Trial
Source: Eur J Pain. 2025 Sep 5;29(9):e70121. doi: 10.1002/ejp.70121 (PMC12412195; doi:10.1002/ejp.70121)
Supplement: Supplementary file 1 — Data S1: ejp70121‐sup‐0001‐supinfo.docx. [file EJP-29-0-s001.docx]

**Table 1.** Description of the multimodal therapeutic intervention program, phase 1.

| Phase 1 | Intervention | Description | Duration |
| --- | --- | --- | --- |
| 1st to 4th Week | 1 | The research participant sits in a chair with the cervical region in a neutral position and performs flexion, extension, inclination, and rotation movements of the cervical region, without load at the maximum possible amplitude. | Three sets of five repetitions for each movement are mentioned. |
|  | 2 | The research participant is in the supine position on the stretcher, with the cervical region resting on the stretcher. The researcher in charge is positioned in front, with fingers in a "bridge" position under the occiput. The head is gently tilted using the radial deviation of the wrist. Then, during the tilt, traction is applied to the cervical region. | Three sustained sets of 10" to 15" of oscillation each. |
|  | 3 | The research participant is in supine position on a stretcher, with the cervical region off the stretcher. The researcher in charge stands in front of the stretcher, places one hand on the occipital region, while the other hand is placed under the mandible. The head was kept in a neutral position. The researcher then tractions the cervical region, leaning backwards using the weight of his own body. | Three sustained sets of 10" to 15" of oscillation each. |
|  | 4 | The research participant is in dorsal decubitus position on a stretcher, with the cervical region outside the stretcher. The researcher in charge stands in front of the stretcher and places his right hand on the occipital region while his left hand is placed under the mandible. The researcher tractions and rotates the cervical region to the left, then to the right, changing the position of the hands. | Three sustained sets of 10" to 15" of oscillation each. |
|  | 5 | The research participant is in dorsal decubitus position on a stretcher, with the cervical region outside the stretcher. The researcher in charge positions his right hand on the occipital region while his left hand is placed under the mandible. The researcher in charge tractions the cervical region with the right hand and consecutively slides the mandible antero-posteriorly with the left hand. | Three sustained sets of 10" to 15" of oscillation each. |
|  | 6 | The research participant is in dorsal decubitus position on the stretcher, with the cervical region outside the stretcher. The researcher in charge stands in front of the stretcher, places his thumbs under the mandible, and makes a lateral slide to the right and left, keeping the head in line with the body to avoid flexion. | Three sustained sets of 10" to 15" of oscillation each. |
|  | 7 | The research participant is in the right lateral decubitus position on the stretcher, with the cervical region resting on the stretcher, the right arm supporting the head, and the left arm in elbow flexion over the body. The researcher in charge is positioned laterally to the stretcher, places one hand on the base of the scapula, and the other stabilizes the participant's shoulder, performing superior/inferior sliding and rotation and retraction movements of the scapula using the movement of the body itself. This intervention should be carried out bilaterally. | Three sets with 30" of oscillation for each movement bilaterally. |
|  | 8 | The research participant is in the supine position on the stretcher, with the cervical region in a neutral position, resting on the stretcher, and arms extended in parallel. The researcher in charge is in front of the stretcher. The participant actively contracts the deep muscles (flexors, extensors, and rotators) of the cervical region, without moving the cervical spine, using eye movements to help perform these exercises. | Three sustained sets of 10" to 15" of oscillation each. |
|  | 9 | The research participant is in dorsal decubitus position on the stretcher, with the cervical region in a neutral position, resting on the stretcher, and their arms extended in parallel. The researcher in charge is in front of the stretcher. The participant performs isometric contractions of the flexor, inclinator, and rotator muscles against the manual resistance of the researcher in charge. | Three sustained sets of 10" to 15" of oscillation each. |
|  | 10 | The research participant, in a prone position on the stretcher, arms flexed and hands resting in front of the chest, with the trunk flexed and the cervical region in a neutral position, contracts the extensors of the cervical spine against gravity. | Three sustained sets of 10" to 15" of oscillation each. |
|  | 11 | The research participant sits in a chair with the cervical region in a neutral position and performs flexion, extension, inclination, and rotation movements of the cervical region, using an elastic band as far as possible, with the researcher supporting the elastic band and positioning himself according to the movement to be performed. | Three sustained sets of 10" to 15" of oscillation each. |

**Table 2.** Description of the multimodal therapeutic intervention program, phase 2.

| Phase 2 | Intervention | Description | Duration | |
| --- | --- | --- | --- | --- |
| 5th to 8th  Week | 1 | The research participant, in the prone position on the mat, with arms extended parallel to the body and knees bent, stabilizes the cervical spine, then anteriorizes the head and returns with the chin tucked in. | | Three sets with 10–15 second holds or 8–12 repetitions, 120 seconds apart. |
|  | 3 | The research participant, on all fours on the mat, maintains support and stabilization of the cervical spine, anteriorizes the head, and returns with the chin tucked in. | | Three sets with 10–15 second holds or 8–12 repetitions, 120 seconds apart. |
|  | 4 | The research participant, on all fours on the mat, stabilizes the cervical spine, then raises the upper limb to 90° alternately, keeping the chin tucked in. | | Three sets with 10–15 second holds or 8–12 repetitions, 120 seconds apart. |
|  | 5 | The research participant lies on all fours on the mat, stabilizing the cervical spine, then raises the upper limb to 90° alternately, and the contralateral lower limb, keeping the chin tucked in. | | Three sets with 10–15 second holds or 8–12 repetitions, 120 seconds apart. |
|  | 6 | The research participant stands, stabilizes the cervical spine, then anteriorizes the head and returns with the chin tucked in. | | Three sets with 10–15 second holds or 8–12 repetitions, 120 seconds apart. |
|  | 7 | The research participant stands, performing a dynamic isometric exercise with an elastic band on the occipital base, with shoulders and elbows in a 90° position, performing elbow extension movement with resistance from the elastic band, keeping the chin tucked in. | Three sets with 10–15 second holds or 8–12 repetitions, 120 seconds apart. | |
|  | 8 | The research participant stands, performing a dynamic isometric exercise with an elastic band on the occipital base, with shoulders and elbows in a 90° position, and executes an elbow extension movement with the resistance of the elastic band on the right, keeping the chin tucked in. | Three sets with 10–15 second holds or 8–12 repetitions, 120 seconds apart. | |
|  | 9 | The research participant stands, performing a dynamic isometric exercise with an elastic band at the occipital base, with shoulders and elbows in a 90° position, performing an elbow extension movement with the resistance of the elastic band on the left, keeping the chin tucked in. | Three sets with 10–15 second holds or 8–12 repetitions, 120 seconds apart. | |
|  | 10 | The research participant stands and performs a dynamic isometric exercise with an elastic band at the base of the occiput, crossed in front, with shoulders and elbows in a 90° position, performing an oblique elbow extension movement to the right and left, keeping the chin tucked in. | Three sets with 10–15 second holds or 8–12 repetitions, 120 seconds apart. | |
|  | 11 | Research participant sitting on a therapeutic ball on a mat. Maintains support and stabilisation of the cervical spine, performs anteriorisation of the head and returns with chin tuck. | Three sets with 10–15 second holds or 8–12 repetitions, 120 seconds apart. | |
|  | 12 | The research participant sits on a therapeutic ball on a mat, stabilizes the cervical spine, then raises the upper limb to 90° alternately, anteriorizing the head and returning with the chin tucked in. | Three sets with 10–15 second holds or 8–12 repetitions, 120 seconds apart. | |
|  | 13 | The research participant sits on a therapeutic ball on a mattress, stabilizes the cervical spine, then raises the upper limb to 90° and the contralateral lower limb alternately, anteriorizes the head, and returns with the chin tucked in. | Three sets with 10–15 second holds or 8–12 repetitions, 120 seconds apart. | |
|  | 14 | The research participant stands with the occipital region supported by the therapeutic ball on a rigid surface, maintaining support and stabilization of the cervical spine, performs head anteriorization, and returns with a chin tuck. | Three sets with 10–15 second holds or 8–12 repetitions, 120 seconds apart. | |
|  | 15 | The research participant stands with the frontal region supported by a therapeutic ball on a rigid surface, maintaining support and stabilization of the cervical spine, performs head anteriorization, and returns to a chin tuck position. | Three sets with 10–15 second holds or 8–12 repetitions, 120 seconds apart. | |
|  | 16 | The research participant stands with the occipital region resting on a therapeutic ball under the surface, performing bilateral shoulder abduction with resistance from an elastic band attached to the feet, keeping the chin tucked in. | Three sets with 10–15 second holds or 8–12 repetitions, 120 seconds apart. | |
|  | 17 | The participant, standing with the frontal region resting on a therapeutic ball under a rigid surface, performs bilateral shoulder abduction with resistance from an elastic band attached to the feet, keeping the chin tucked in. | Three sets with 10–15 second holds or 8–12 repetitions, 120 seconds apart. | |
|  | 18 | The participant stands, stabilizing the cervical region, performs a dynamic lifting movement of the upper limbs, with a therapeutic ball, associated with flexion of the lower limbs, keeping the chin tucked in. | Three sets with 10–15 second holds or 8–12 repetitions, 120 seconds apart. | |
|  | 19 | The participant stands with cervical stabilization, performing functional dynamic exercises with scapular adduction and shoulder external rotation, using resistance from elastic bands crossed over the palms of the hands. | Three sets with 10–15 second holds or 8–12 repetitions, 120 seconds apart. | |
|  | 20 | The participant stands one step forward, maintaining cervical stabilization, and performs functional dynamic exercises with bilateral shoulder extension and scapular retraction using elastic band resistance. The researcher in charge is in front of the participant, stabilizing the elastic band. | Three sets with 10–15 second holds or 8–12 repetitions, 120 seconds apart. | |
|  | 21 | The participant stands one step forward, maintaining cervical stabilization, and performs functional dynamic exercises with bilateral shoulder elevation and scapular protraction using elastic band resistance. The researcher in charge is behind the participant, stabilizing the elastic band. | Three sets with 10–15 second holds or 8–12 repetitions, 120 seconds apart. | |
|  | 22 | The participant stands one step forward, maintaining cervical stabilization, and performs a functional dynamic exercise of shoulder abduction and bilateral elbow extension with scapular retraction, using resistance from an elastic band crossed over the palms of the hands. | Three sets with 10–15 second holds or 8–12 repetitions, 120 seconds apart. | |
|  | 23 | The participant stands with bipodal support, maintaining cervical stabilization, and performs a functional dynamic exercise of bilateral shoulder elevation and elbow extension with resistance from an elastic band crossed over the chest. | Three sets with 10–15 second holds or 8–12 repetitions, 120 seconds apart. | |
|  | 24 | The participant stands with bipodal support, maintaining cervical stabilization, and performs a functional dynamic exercise of bilateral shoulder elevation, elbow extension, and wrist pronation with resistance from an elastic band crossed over the chest. | Three sets with 10–15 second holds or 8–12 repetitions, 120 seconds apart. | |
